# Supplementary material for: WordCommentsAnalyzer: A windows software tool for qualitative research
Source: F1000Res. 2018 Sep 4;7:536. Originally published 2018 May 3. [Version 2] doi: 10.12688/f1000research.14819.2 (PMC6137411; doi:10.12688/f1000research.14819.2)
Supplement: Supplementary file 3 [file f1000research-7-17614-s0002.tgz › b2a79496-4ada-47c4-9682-a99ce867836d.zip!/Eight Iranian Health Journals/Yafteh _34.docx]

© Yafteh  . This work is licensed under CC BY 4.0 (https://creativecommons.org/licenses/by/4.0/).

Volume & Issue: دوره 12 - شماره 2

Title: عدالت در توزیع تخت های مراقبت ویژه در ایران از رویکرد منحنی لورنز و ضریب جینی

http://yafte.lums.ac.ir/article-1-317-fa.html

دسترسی برابر به خدمات بهداشتی و درمانی از حقوق اساسی انسان ها است. توزیع نامتوازن منابع بیمارستانی به ویژه تخت های مراقبت ویژه، دسترسی برابر افراد به این مراقبت های حیاتی را با مشکل مواجه می نماید تخت های مراقبت ویژه یکی از مهمترین و با ارزش ترین منابع سیستم بهداشت و درمان بوده که عدم تخصیص برابر و عادلانه آن در بین استان های کشور ممکن است علاوه بر جابجایی بیماران بدحال منجر به عوارض جبران ناپذیر گردد. هدف از این مطالعه بررسی میزان عدالت در توزیع تخت های CCU1, ICU2, Post CCU3, Post ICU4, NICU5 بین استان های کشور بود. مواد و روش ها: مطالعه حاضر از نوع مطالعات کاربردی بوده که به روش توصیفی-تحلیلی نگاشته شده است، داده های مورد نیاز از آخرین آمارهای مستند موجود در وزارت بهداشت، درمان و آموزش پزشکی و مرکز آمار ایران جمع آوری گردیده است. میزان عدالت توزیعی با استفاده از پرکاربردترین شاخص های آماری در این زمینه یعنی منحنی لورنز و ضریب جینی از طریق نرم افزار Excel 2007 محاسبه شد. همچنین نقشه جغرافیایی تراکم تخت های مراقبت ویژه در کشور نیز ترسیم گردید که قطعا برای سیاست گذاری مناسب در حیطه سلامت ضروری می باشد. بحث و نتیجه گیری: اگرچه نقشه های جغرافیایی تراکم تخت های مراقبت ویژه، تراکم متفاوت این تخت ها در استان های کشور را نشان می دهد ولی این اختلاف از نظر شاخص جینی قابل اغماض بوده و در نهایت می توان نتیجه گرفت تقریبا برابری کامل در توزیع تخت های مراقبت ویژه (بجز تخت های NICU) در میان استان های کشورمان رعایت گردیده. یافته ها: تعداد کل تخت های NICU, Post ICU, Post CCU, ICU, CCUدر کشور به ترتیب 4194، 3720، 4097، 291، 1129 بود. بیشترین مقادیر برای هریک از تخت های مراقبت ویژه موجود در هریک از استان ها، در استان تهران گزارش شد. بیشترین نسبت تخت های NICU, Post ICU, Post CCU, ICU, CCU به ازای هر 100,000 نفر جمعیت به ترتیب در استان های یزد ، تهران ، یزد ، قم ، خراسان رضوی مشاهده شد . در پایان ضریب جینی برای تخت های NICU, Post ICU, CCU, ICU, CCU Post به ترتیب 13/0 ،17/0، 15/0، 11/0، 23/0 محاسبه گردید.

Title: بررسی میزان شیوع هیپوتیروئیدی درکودکان تحت درمان با داروهای ضدتشنج مراجعه کننده به بیمارستان شهید مدنی خرم آباد در نیم سال دوم 1387

http://yafte.lums.ac.ir/article-1-316-fa.html

هیپوتیروئیدی از جمله مشکلاتی است که در دوره شیرخوارگی و کودکی می تواند باعث اختلال شدید در رشد و تکامل آنان گردد. از عواملی که می تواند هیپوتیروئیدی را در کودکان ایجاد کند افزایش متابولیسم کبدی T4 و T3 است که به دنبال افزایش فعالیت آنزیم های میکروزومال کبدی و مصرف داروهای ضد تشنج مثل فنوباربیتال، کاربامازپین، سدیم والپرات و فنی توئین دیده می شود. هدف از این مطالعه بررسی میزان شیوع هیپوتیروئیدی در کودکان تحت درمان با داروهای ضد تشنج مراجعه کننده به درمانگاه بیمارستان شهید مدنی خرم آباد در نیم سال دوم 1387 می باشد. مواد و روش ها: در این مطالعه نمونه از میان کودکانی تهیه گردید که تحت درمان با داروهای ضد تشنج بودند. معیارهای پذیرش نمونه مانند سن بین 3 ماه تا 13 سال، مصرف داروهای ضد تشنج حداقل به مدت دو ماه، داشتن یک EEG غیر نرمال بوده و در نیم سال دوم 1387 به درمانگاه بیمارستان شهید مدنی خرم آباد مراجعه کرده باشند. اجزای جمع آوری اطلاعات شامل پرسشنامه حاوی اطلاعات شخصی بیمار مانند سن، جنس، نوع داروی مصرفی، مدت زمان مصرف دارو بودند. پس از جمع آوری اطلاعات آزمایشهای T4 ، TSH و T3RU برای بیماران انجام گرفت و تجزیه و تحلیل شد. یافته ها: در بازه زمانی3 تا 13 سال 38 کودک مبتلا به صرع که داروهای ضدتشنج مصرف می کردند بررسی گردیدند، 4 بیمار (5/10 درصد) هیپوتیروئید بودند که 2 بیمار (25/5 درصد) به هیپوتیروئیدی کلینیکال و 2 بیمار (25/5 درصد) به هیپوتیروئیدی ساب کلینیکال مبتلا بودند. از 4 کودک بیمار 2 بیمار دختر و 2 بیمار پسر بودند. میانگین سنی 2 5/6 سال با میانه 5/6 سال بود. هیپوتیروئیدی در این کودکان تفاوت معنی داری بود. بحث و نتیجه گیری: میزان شیوع هیپوتیروئیدی در کودکان تحت درمان با داروهای ضد تشنج مراجعه کننده به درمانگاه بیمارستان شهید مدنی خرم آباد در نیم سال دوم 1387، 5/10 درصد بود. هم چنین بین سن و جنس بیماران تحت درمان با داروهای ضد تشنج و ابتلاء به هیپوتیروئیدی ارتباط معنی داری وجود نداشت. بین مدت مصرف داروهای ضد تشنج و ابتلا به

Title: اثر یک برنامه کاهش وزن بر کارکرد ریوی و التهاب سیستمیک در مردان چاق

http://yafte.lums.ac.ir/article-1-314-fa.html

التهاب سیستمیک با کاهش کارکرد ریوی در افراد چاق همراه است. اینکه تمرین هوازی در افراد چاق با بهبود التهاب سیستمیک و کارکرد ریوی همراه است روشن نمی‌باشد. هدف مطالعه حاضر بررسی اثر 12 هفته تمرین هوازی بر سطح سرمی پروتئین واکنشگر C و عملکرد ریوی در افراد چاق بود. بحث و نتیجه گیری: تمرین هوازی منجر به بهبود شاخص‌های چاقی و عملکرد ریوی در مردان چاق می‌شود و این بهبودی با سطوح کاهش یافته پروتئین واکنشگر C همراه است یافته ها: در سطح پایه افراد چاق نسبت به افراد با وزن طبیعی دارای سطوح بالاتر پروتئین واکنشگر C و کارکرد ریوی ضعیف تر بودند (05/0p<). بعد از 12 هفته تمرین هوازی وزن بدن، دور کمر، چربی احشایی، کل چربی شکمی و پروتئین واکنشگر C کاهش یافت (05/0 p <). در مقابل، شاخص‌های عملکرد ریوی متعاقب برنامه تمرین هوازی بهبود یافت (05/0 p <). مواد و روش ها: بیست و سه مرد چاق (سن: 9/3±19/46 سال، نمایه توده بدنی: 87/0±64/31 کیلوگرم بر متر مربع) به طور تصادفی به گروه‌های تمرین هوازی (12 نفر) و کنترل (11 نفر) تقسیم شدند. همچنین یک گروه کنترل با وزن طبیعی (9 نفر) برای مقایسه در سطح پایه فراخوانده شد (سن: 1/4±36/45 سال، نمایه توده بدنی: 45/0±63/24 کیلوگرم بر متر مربع). برنامه تمرین هوازی سه جلسه در هفته به مدت 12 هفته بود. ترکیب بدنی (سی تی اسکن)، شاخص‌ التهابی و کارکرد ریوی قبل و بعد از مداخله اندازه‌گیری شد.

Title: بررسی دیدگاه اعضای هیات علمی در خصوص بعضی از شاخص های آموزشی در دانشگاه علوم پزشکی لرستان 1387

http://yafte.lums.ac.ir/article-1-313-fa.html

مواد و روش ها: در این مطالعه توصیفی، تحلیلی از پرسشنامه ای مشتمل بر دو قسمت (مشخصات دموگرافیک اعضای هیات علمی و نظرهای آنان در خصوص شاخص های آموزشی) استفاده گردید. برای تعیین روایی از روایی محتوا و برای تعیین پایایی ابزار از یک مطالعه مقدماتی و تعیین آلفای کرانباخ استفاده شد که ضریب همبستگی 81/0 را نشان داد. برای تجزیه و تحلیل داده ها از آمار توصیفی و آمار تحلیلی (غیر پارامتری ، اسپیرمن کروسکال والیس ، منویتنی آزمون کای دو) spss ver باتوجه به اینکه در شرایط زندگی امروز، نیازهای جامعه در حال تغییر دایمی هستند، چنین توصیه شده است که ما نیز می بایست در آموزش خود تغییر ایجاد کنیم. نظر به اینکه استفاده از تجربیات مدرسان می تواند نقش مهمی در این خصوص داشته باشد ، در این راستا مطالعه حاضر با هدف تعیین دیدگاه اعضای هیات علمی در مورد بعضی از شاخص های آموزشی در دانشگاه علوم پزشکی لرستان در سال 1387 صورت گرفت. بحث و نتیجه گیری: 9% اعضای هیات علمی، کارگاههای روش تدریس را مناسب با نیازهای آموزشی خود ندانستند ، امااکثریت برگزاری کارگاههایی را به شکل مداوم و متوالی ضروری دانسته اند. همچنین اعضای هیات علمی میزان توجه مستولین به مشکلات آموزشی و هماهنگی رشته تحصیلی با نیاز جامعه ، تصمیمم گیری مشارکتی میزان تناسب تعداد دانشجویان با امکانات و فضای آموزشی و نیروی انسانی را خواستار شدند.. چنین به نظر می رسد که توجه زیاد مسئولین به کمیت و کیفیت کارگاهها و آموزش مداوم و رفع مشکلات آموزشی دانشگاه قابل تاکید است و استفاده از مشارکت بیشتر اعضای هیات علمی در برنامه ریزی آموزشی و مدیریتی توصیه می گردد. یافته ها: از مجموعه اعضای هیات علمی شاعل در دانشگاه، 100 نفر پرسشنامه را تکمیل نمودند. 59% آنان مذکر، محل اشتغال 45% در دانشکده پزشکی، 76% در کارگاههای روش تدریس شرکت کرده بودند و 66.9% کارگاه را با نیازهای آموزشی مدرسان مناسب دانستند. همچنین گذراندن دوره یا واحد خاصی را در خصوص روش تدریس (91% )، برنامه ریزی آموزشی 90 % را برای مدرس الزامی و زمان مناسب آنرا قبل از شروع بکار دانسته اند. مهمترین شاخصهای آموزشی داشتن توان علمی( 82% ) ، هماهنگی رشته تحصیلی با نیاز جامعه ( 73% ) ، مشارکت اساتید در برنامه ریزی آموزشی (72 %) ، تناسب کارگاه با نیاز آموزشی مدرس (62%) ، عدم هماهنگی رشته های آموزشی با نیاز جامعه (54%)، تناسب تعداد دانشجویان با امکانات و فضای آموزشی و نیروی انسانی (68 % ) را بسیار با اهمیت دانستند.

Title: مقایسه تاثیر انتونوکس و آب گرم بر درد زایمان

http://yafte.lums.ac.ir/article-1-312-fa.html

درد زایمان یکی از شدیدترین دردهایی است که زنان تجربه می کنند.از علل عمده سزارین انتخابی ، ترس از درد زایمان است. انتونوکس را مفیدترین انتخاب برای کاهش درد و بی خطرترین روش برای مادر و جنین می دانند. استفاده از وان آب گرم نیز برای کاهش درد آرامش و راحتی مادر در طی زایمان مفید است. هدف از این پژوهش تعیین مقایسه اثر انتونوکس و آب گرم بر درد زایمان بود. مواد و روش ها: این مطالعه کار آزمایی بالینی در زایشگاه عسلی خرم آباد و در سال1386 انجام شد.که نمونه ها،150 نفر زن باردار در فاز فعال مرحله اول زایمان با داشتن معیارهای پذیرش نمونه انتخاب وبراساس نمونه گیری آسان و تخصیص تصادفی واحدها به سه گروه 50 نفری تقسیم شدند.گروه تجربی اول از انتونوکس، گروه تجربی دوم از آب گرم و گروه کنترل از روش روتین زایشگاه استفاده کردند. در ابتدا نمره درد در طی سه انقباض و مجدد پس از اجرائ روش کاهش درد نمره درد درطی سه انقباض تعین گشت. برای تعیین نمره درد از مقیاس درجه ای آنالوگ بینایی استفاده شد. درحین اجرائ روشها از پالس اکسی متر جهت تعین شدت تغییرات ضربان قلب و میزان اشباع اکسیژن خون SaO2 مادر در پاسخ به درد استفاده می شد. یافته ها: میانگین نمره درد در روش انتونوکس از روش آب گرم بطور معنی دار (001/0p<) پایینتر بود. پیشرفت زایمان در روش انتونوکس از روش آب گرم بیشتر بود. این اختلاف با (005/0p=) معنی دار بود. میانگین افزایش نبض مادر در پاسخ به درد در روش انتونوکس از روش آب گرم کمتر بود. این اختلاف با (001/0p<) معنی دار بود. میانگین SaO2 مادران مورد پزوهش در روش انتونوکس از روش آب گرم بطور معنی دار (001/0p<) بیشتربود. بحث و نتیجه گیری: این برسی نشان می دهد که گاز انتونوکس در مقایسه با روش آب گرم تاثیر بیشتری در کاهش درد زایمان دارد و نیز موجب پیشرفت بهتر زایمان و نیز اکسیژن رسانی بهتر به مادر و جنین به دلیل کاهش بیشتر درد می شود.

Title: عوامل مؤثر بر خشونت علیه زنان در استان لرستان

http://yafte.lums.ac.ir/article-1-308-fa.html

خشونت علیه زنان یک پدیده جهانی است که روی سلامت و بهداشت روانی خانواده و جامعه تاثیر منفی می گذارد. از طریق شناخت به میزان و نوع خشونت و عوامل مؤثر بر آن می توان در جهت پیشگیری و کاهش آن ، اقدامات درمانی و مداخله جویانه مطلوب را انجام داد. هدف تحقیق ، شناسایی انواع خشونت و میزان شدت آن در خانواده و همچنین شناسایی عوامل زمینه ای موثر بر خشونت علیه زنان در استان لرستان می باشد. مواد و روش ها: این مطالعه یک پژوهش توصیفی است که با استفاده از روش همبستگی انجام گرفته است. حجم نمونه آماری بر اساس فرمول کوکران و با توجه به جمعیت جامعه 383 نفر در نظر گرفته شد. واحد تحلیل بر اساس ماهیت مسئله ، خانواده و سطح مشاهده زنان متاهل است.جمع آوری اطلاعات از طریق پرسشنامه صورت گرفت و شامل : 1- سئوالات مربوط به متغیرهای جمعیت شناختی 2- پرسشنامه محقق ساخته در مورد زمینه های خطرساز و تصور از نقش های جنسیتی 3- خشونت علیه زنان حاج یحیی سال 1999 بود. در تحلیل داده ها نیز از روش ضریب همبستگی پیرسون و تحلیل واریانس یک طرفه استفاده شد. بحث و نتیجه گیری: به طور کلی نتایج تحقیق نشان داد که سن زنان ، سطح سواد ، جمعیت خانواده و شغل همسر با خشونت علیه زنان رابطه دارد . همچنین رفتار پرخاشگرانه والدین در دوران کودکی در خشونت علیه زنان نقش دارد. ازسوی دیگر تصور سلطه گرایانه مرد نسبت به زن در بروز پرخاشگری مؤثر است. یافته ها: یافته های این بررسی نشان می دهد که در بین انواع خشونت ، خشونت روانی و جسمی بیش از سا یر اشکال آن در زنان تجربه می شود. بین خشونت خانگی و متغیر سن ، سطح سواد ، تعداد فرزندان و شغل همسر رابطه معناداری دیده شد اما نوع ازدواج ،درآمد همسر و سن همسر رابطه معناداری با خشونت خانگی نشان نداد. همچنین میزان خشونت در خانواده های گسسته بیشتر مشاهده شد و بین وجود الگوی پرخاشگری در خانواده پدری در دوران کودکی و بروز خشونت رابطه وجود داشت . از سوی دیگر رابطه معناداری بین تصور سلطه گرایانه مرد و خشونت خانگی دیده شد .

Title: استفاده از کانال راهنمای عصب در ترمیم عصب محیطی

http://yafte.lums.ac.ir/article-1-309-fa.html

اگر چه از اتوگرافت عصب به عنوان روش استاندارد طلایی بالینی جهت ترمیم شکاف های عصبی استفاده می شود ، پیشرفت های زیادی جهت هدایت آکسون های رژنره شده پس از اسیب عصب انجام شده است. بهبود فانکشنال بعد از آسیب عصب محیطی به رژنراسیون صحیح آکسون ها در جهت بافت های هدف اولیه انها وابسته است. با افزایش چشم انداز های رژنراسیون اکسونی و بهبود فانکشنال آن ، تحقیقات بر روی طراحی کانال های راهنمای عصب یا NGCs متمرکز شده است. NGCs مجاری لوله ای شکل از مواد طبیعی یا مصنوعی هستند ،که به عنوان پل ارتباطی بین دو انتهای عصب اسیب دیده استفاده می شوند. این مقاله مروری رژنراسیون عصب محیطی را در کانال راهنمای عصب شرح می دهد.
